# Assessing environmental and management factors that drive soybean yield gaps in Brazil

**DOI:** 10.1002/jeq2.70076

**Published:** 2025-09-09

**Authors:** Mauricio Fornalski Soares, Gean Leonardo Richter, Nereu Augusto Streck, Fabio Ricardo Marin, Evandro Henrique Figueiredo Moura da Silva, Eduardo Lago Taglieapietra, José Eduardo Minussi Winck, Michel Rocha da Silva, Felipe Schmidt Dalla Porta, Júlia Farias, Alencar Junior Zanon

**Affiliations:** ^1^ Department of Crop Science Federal University of Santa Maria Santa Maria Brazil; ^2^ Crops Team–Consulting, Research and Development Santa Maria Brazil; ^3^ Paulo/ESALQ ‐ Department of Biosystems Engineering University of São Paulo Piracicaba Brazil; ^4^ The Biodesign Institute Center for Environmental Health Engineering Arizona State University Tempe Arizona USA

## Abstract

Brazil is the world's largest producer and exporter of soybeans (*Glycine max* L. Merr.). Assessing yield gaps (Yg) is essential for improving resource use efficiency and guiding farmers’ management strategies. The objective of this study was to estimate soybean yield potential (Yp), water‐limited yields (Yw), and Yg based on water and agricultural practices across Brazil's five soybean macroregions. We have quantified yield losses due to delayed sowing and evaluated interannual yield variability caused by environmental and climatic factors. The results revealed that the southern regions had the highest Yp values but also the largest Yg values, which were strongly influenced by climatic factors. In contrast, the Brazilian Midwest had the lowest Yp yet minimal water‐related Yg, with relatively stable yields over time; here, Yg were primarily due to crop management rather than climatic constraints. In northern macroregions, lower Yp was observed with moderate climatic influences. Delayed sowing reduced Yp across all macroregions, with the greatest losses occurring in regions with initially high Yp, particularly in the south. Each macroregion has unique environmental conditions that lead to different patterns of Yp, Ya (actual yield), and Yw. In the southern macroregions, Yg are primarily due to water constraints, indicating potential benefits of irrigation, while the Midwest, which has the lowest Yg, improved crop management practices offer the most significant opportunity for yield gains.

## Core Ideas

- Soybean is a main crop for the global protein supply.
- Identifying soybean yield potential allows for increased yield without expanding area.
- Covering management and environmental yield gaps leads to food security.
- Adjusting the sowing date of soybeans mitigates water deficit losses.
- Climate change impacts soybean yield differently in distinct environments.

## Plain Language Summary

Soybeans are an important crop worldwide, and Brazil is its leading producer. But in many regions, farmers are harvesting much less than they could. This study looked at five key soybean regions in Brazil to determine how much yield is occurring and why. We found that in the south, drought is the main reason for lower yields, while in the central and northern regions, better crop management could significantly increase production. Sowing soybeans later than ideal also reduced the yield potential in all regions. These results show that strategies such as adjusting sowing dates, improving cultivation methods, or investing in irrigation can increase soybean yields. By closing yield gaps, Brazil can grow more food without expanding acreage, which promotes both food security and environmental sustainability.

**Abbreviations**
CV: coefficient of variation
CZ: climatic zone
ENSO: El Niño‐Southern Oscillation
GYGA: Global Yield Gap Atlas
K–S: Kolmogorov–Smirnov test
MG: maturity group
MR: Macroregion
NASA‐POWER: NASA prediction of worldwide energy resources
RWS: reference weather station
Ya: actual yield
Yg: yield gaps
Ygm: management yield gap
Ygw: water yield gap
Yp: yield potential
Yw: water‐limited yields

## 1 INTRODUCTION

Closing yield gaps (Yg) is a central challenge for ensuring food security, especially in major agricultural nations like Brazil. The Yg can be defined as the difference between farmers’ actual yield (Ya) and the yield potential (Yp), where Yp is the yield attainable under optimal agronomic management with minimal losses from biotic and abiotic stresses, and it is a key biophysical indicator of the available room for crop production increase with current land and water resources (Rattalino Edreira et al., 2021). Marin et al. (2022) estimated the usable Yg as the difference between the achievable yield and the average yield and found that the exploitable Yg for soybean (*Glycine max* L. Merr.) in Brazil increases northward and projected that if Brazil were to close its exploitable Yg, soybean production could increase by 162 million tonnes without deforestation and with 58% lower greenhouse gas emissions compared to current trends. Global studies of staple crops such as rice (*Oryza sativa* L.), wheat (*Triticum aestivum* L.), maize (*Zea mays* L.), and soybean demonstrate that achieving high yields is compatible with high resource‐use efficiency when supported by precision management and sustainable intensification strategies (Cassman & Grassini, 2020).

Brazil is currently the world's largest soybean producer and exporter, accounting for nearly 40% of global supply (USDA Foreign Agricultural Service, 2025). Soybean cultivation in Brazil spans >4000 km, from latitudes 3°N to 33°S, across a broad range of tropical and subtropical climates, soil types, and microclimates. This geographic diversity results in pronounced regional differences in yield potential, yield stability, and production constraints. To account for this diversity, the Brazilian Ministry of Agriculture, Livestock and Supply (MAPA) has adopted a framework that divides the country into five soybean macroregions (MRs) based on latitude, altitude, soil types, and climate conditions (Kaster & Farias, 2012). These macroregions serve as a basis for cultivar recommendation, research, and management strategies tailored to regional characteristics. Examining Yg within this macroregional framework allows for more precise identification of constraints and targeted interventions that can enhance productivity while reducing environmental impacts.

Some soybean‐producing regions in Brazil achieve high Yp but are highly sensitive to rainfall variability and El Niño Southern Oscillation (ENSO) events, which reduce water‐limited yields (Yw). Identifying these climatic patterns is crucial for developing management strategies, such as adjusting sowing dates and adopting irrigation systems tailored to regional conditions, to mitigate yield losses. Previous studies in Brazil reported Yp values ranging from 5.6 to 11.3 Mg ha^−1^, but methodological differences, such as the lack of climate zoning and process‐based crop models, limit comparability. Therefore, revising soybean Yp estimates using standardized approaches like the Global Yield Gap Atlas (GYGA, https://www.yieldgap.org) is necessary for robust regional and international comparisons.

Given the importance of soybeans for global food and protein supply, and Brazil's role as a major producer, closing Yg offers significant economic and environmental benefits. This study aimed to (i) estimate the Yp and Yw for the five soybean MRs in Brazil using the standardized GYGA framework, (ii) decompose the Yg into its water‐ and management‐related components, (iii) evaluate regional yield losses associated with delayed sowing dates, and (iv) analyze interannual yield variability driven by environmental and climatic factors in each MR. Together, these analyses provide a foundation for regionally tailored strategies that can support sustainable intensification and climate‐resilient soybean production in Brazil.

## 2 MATERIALS AND METHODS

### 2.1 Brazilian soybean MRs

**FIGURE 1:**
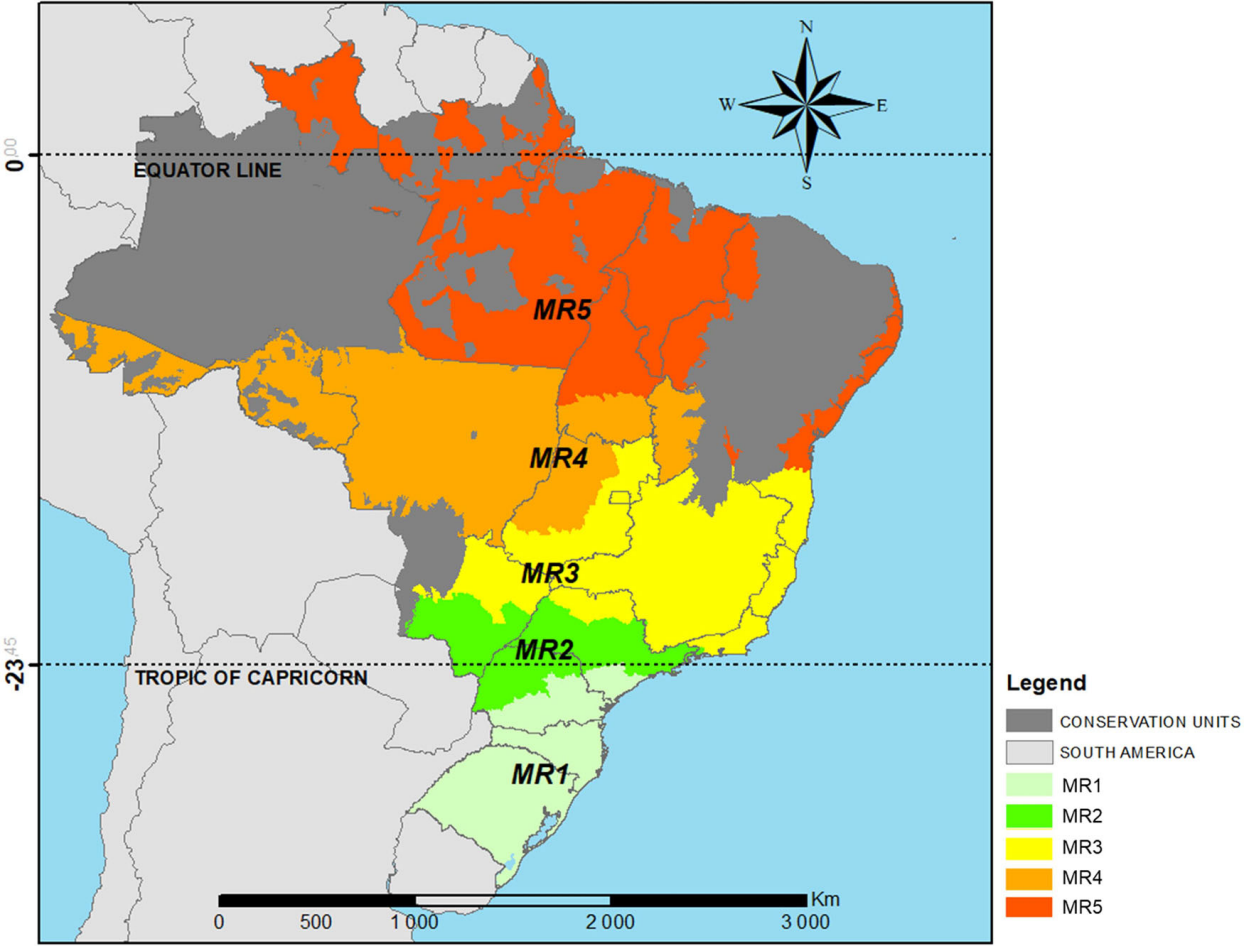
Brazilian soybean growing macroregions.

Considering the diversity of ecosystems and soil and climate types (latitude and altitude) in the country, Embrapa Soja submitted a proposal to the MAPA to regionalize the indication of soybean cultivars for Brazil. Subsequently, researchers from different institutions aided in improve the proposal, resulting in the model being approved by MAPA (third approximation), according to Kaster and Farias (2012). Five MRs for soybeans were established for research and cultivar indication (Figure 1). This classification into MRs allows a visualization of the different soybean production regions in Brazil, making it possible to carry out stratified evaluations and adopt management practices or targeted public policies according to their needs and specificities.

Macroregion 1 (MR1) includes soybean‐growing areas in the states of Rio Grande do Sul, Santa Catarina, Paraná, and part of São Paulo, which account for 24.6% of Brazil's soybean harvest area. The range of MG's is between 5.0 and 7.7, and sowing is recommended from September 9° to January 8°. Macroregion 2 (MR2) comprises the states of Paraná, São Paulo, and Mato Grosso do Sul and accounts for 18.0% of the soybean area in Brazil. The regular MG for this MR is 6.0–7.0 and sowing takes place from September 11° to December 24°. Macroregion 3 (MR3) comprises the states of Mato Grosso do Sul, São Paulo, Minas Gerais, and Goiás and accounts for 14.4% of the soybean harvest area. The MG used are between 6.0 and 8.7, with sowing taking place between September 16° and January 8°. Macroregion 4 (MR4) includes Acre, Rondônia, Mato Grosso, Bahia, Goiás, and Tocantins, with 35.9% of the Brazilian soybean area. The MGs used range from 7.0 to 9.5, and the sowing dates are from September 1° to February 24°. Finally, macroregion 5 (MR5) comprises the northern and northeastern states of Piauí, Maranhão, Tocantins, Pará, Roraima, Bahia, Sergipe, Alagoas, and Amapá and accounts for 7.0% of the Brazilian soybean area. The MG range is from 7.7 to 10.0, and the recommended sowing dates are between September 16° and June 26°.

### 2.2 Data sources and assumptions

Data source selection and quality control followed the guidelines of the GYGA (Rattalino Edreira et al., 2021). Harvested area and average yields (Ya) for 2017–2022 crop seasons were retrieved from the Brazilian Institute of Geography and Statistics (IBGE). It should be noted that for the application of the GYGA method, the Ya series from 2012 to 2017 was used, and Ya data from 1999 to 2019 were used to evaluate the impact of the ENSO phenomenon on the interannual yields of the same regions. Reference weather stations (RWS) were selected based on crop area distribution and agroclimatic zones (CZ) defined for Brazil, where each CZ corresponds to a combination of growing degree days, aridity index, and temperature seasonality (Van Wart et al., 2013).

**Core Ideas**

- Soybean is a main crop for the global protein supply.
- Identifying soybean yield potential allows for increased yield without expanding area.
- Covering management and environmental yield gaps leads to food security.
- Adjusting the sowing date of soybeans mitigates water deficit losses.
- Climate change impacts soybean yield differently in distinct environments.

Daily maximum and minimum temperatures and precipitation for the period from 1999 to 2019 were retrieved from the Brazilian Institute of Meteorology. Relative humidity, dew point temperature, and evapotranspiration were estimated following Allen et al. (1998). Solar radiation was retrieved from NASA's prediction of worldwide energy resources (NASA‐POWER) (Bender & Sentelhas, 2018; Duarte & Sentelhas, 2019; Monteiro et al., 2018). Quality control and padding/correction of weather data were performed based on the propagation technique developed by Van Wart et al. (2013). Measured weather data were not available in 20% of the buffers; therefore, we used weather data (including all variables) from NASA‐POWER. Prior to their use, the correlation between NASA‐POWER data and ground‐based weather station data were evaluated, showing good agreement for key variables such as solar radiation and temperature (Mourtzinis et al., 2017; Van Wart et al., 2013). These studies demonstrated that, when validated, gridded weather datasets like NASA‐POWER can reliably complement measured data to fill gaps or cover areas with no local records, without compromising the precision of crop yield simulations. The long‐term (20 years) daily weather used was suitable for robust estimation of average Yw and its variability (Grassini et al., 2015).

The predominant soil series were identified for each RWS buffer based on data from the Radambrasil project (Cooper et al., 2005). Soils were selected to cover at least 30% and at least 10% of the area in each buffer. The selected soils were confirmed by local experts and modified as needed to ensure that the simulated soils represented the most common agricultural soils in each buffer. Rooting depth was set at 0.8 m to account for root growth limitation in deep horizons due to low pH and differences among plant species in rooting patterns and/or tolerance to low pH (Battisti et al., 2017; Franchini et al., 2017; Pivetta et al., 2011). Calibrated pedotransfers functions for tropical soils were used to derive soil water limits (Tomasella & Hodnett, 2004). Field capacity was set at −10 kPa, following observations for tropical soils by Reichardt and Timm (2020) and Tomasella and Hodnett (2004).

Management for each RWS buffer zone was obtained from local agronomists from EMBRAPA and other experts. Information included crop rotations and the proportion of each crop in the total harvested area, the sowing window, the name of the predominant cultivar and MG, and the optimal plant density (CONAB, 2020). To calculate the yield losses due to the delay in sowing date, daily simulations were performed in all buffer zones classified according to the five soybean MRs. A detailed list of weather stations, associated management practices, and soil types used for Decision Support System for Agrotechnology Transfer—CROPGRO (crop growth model) simulations is provided in the Supporting Information. This includes information on sowing dates, maturity groups (MG), plant populations, and the predominant soil types and their relative proportions within each buffer zone. These data enhance the transparency and reproducibility of the modeling framework, and further highlight the robustness of the dataset and assumptions used in the simulations.

Following Van Bussel et al. (2015), each combination of soil types was weighted by its relative contribution in the RWS buffer to calculate the average Yw and Yp values. For all Yp, Yw, and Ya in each RWS buffer, the water yield gap (Ygw) was calculated as the difference between Yp and Yw, and the management yield gap (Ygm) was calculated as the difference between Yw and Ya. Model calibration and evaluation for the region under study has been done previously by Tagliapietra et al. (2021) and Ribas et al. (2021).

The photothermal coefficient (*Q*) was calculated by dividing the solar radiation by the average air temperature determined in the model for the period from R1 to R7 minus the base temperature (Fischer, 1985), assuming a base temperature of 0°C for the reproductive phase (Setiyono et al., 2007). The Yp line was calculated using the average of the 5% yields that were below the highest yield, and the yield loss (slope of the straight line) was calculated using the linear equation obtained from the remaining values. The date of onset of yield loss is the value corresponding to the union of the two curves (Zanon et al., 2016).

### 2.3 Statistical approach

All datasets were subjected to basic and descriptive statistics, calculating location (arithmetic mean and median) and dispersion (standard deviation, variance, and coefficient of variation [CV]), as well as coefficients for skewness and kurtosis, which relate to the characteristics of the data distribution. The Kolmogorov–Smirnov (K–S) test with a significance level of 5% was applied to check the normality of all datasets.

The nonparametric method of Kruskal–Wallis test was applied to determine whether the modeled data of Yp and Yg were the same or different in the five MRs evaluated. When the value of the Kruskal–Wallis statistic is calculated to be statistically significant, it means that at least one of the compared groups is different from the others. The corresponding two‐sided procedure of pairwise multiple comparison, the Nemenyi test, is based on common rankings, where sample data from all populations are ranked together (Liu & Chen, 2012). The Nemenyi test is like the Tukey test for analysis of variance and was used to compare the modeled variables from the five MRs. Scatterplots, linear regression plots, pie charts, boxplots, and scatterplots with calculation of CV were also used to generate the figures. All statistical methods were performed using R (R Core Team, 2023).

## 3 RESULTS

### 3.1 Dataset exploratory analysis

Table 1 summarizes the descriptive statistics for Yp, Yw, actual yield (Ya), and the yield gaps attributed to water (Ygw) and suboptimal management (Ygm). Across Brazil, the mean Yp was 6.66 Mg ha^−1^, ranging from 5.68 to 7.52 Mg ha^−1^. The mean Yw was 5.47 Mg ha^−1^ (3.12–6.94 Mg ha^−1^), indicating a significant water‐related Yg at the national scale. The mean Ya was only 3.00 Mg ha^−1^, representing <50% of Yp and highlighting considerable room for improvement through optimized management practices and technologies. Based on the classification by Wilding and Drees (1983), the coefficients of variation (CV) for Yp and Ya were low (CV ≤ 15%), while Yw and Ygm showed moderate variability (15% < CV ≤ 35%). In contrast, Ygw exhibited high variability (CV > 35%). Skewness (−1.30 for Ya and 0.89 for Ygw) and kurtosis values (1.74 and 0.90, respectively) suggest that national productivity and water limitations are the most variable factors across space and time.

**TABLE 1:** Output dataset exploratory analysis.

Var.	Mean	Med	Min	Max	SD	CV (%)	Skew	Kurt	K–S
Yp	6.66	6.73	5.68	7.52	0.48	7.3	−0.30	−0.55	0.17^N^
Yw	5.47	5.53	3.12	6.94	0.90	16.5	−0.66	0.13	0.34^N^
Ya	3.00	3.06	2.33	3.35	0.23	7.5	−1.30	1.74	0.01
Ygw	1.20	0.76	0.00	3.80	1.09	90.8	0.89	−0.90	0.01
Ygm	2.46	2.51	0.79	3.80	0.79	32.2	−0.42	−0.50	0.68^N^

Abbreviations: CV (%), coefficient of variation; Kurt, coefficient of kurtosis; K–S Kolmogorov–Smirnov test (normality = *p*‐value > 5%); Max, maximum value; Mean, mean value; Med, median value; Min, minimum value; N, follow normal distribution; SD, standard deviation; Skew, coefficient of skewness; Var., variables; Ya, yield average; Ygm, yield gap caused by suboptimal crop management; Ygw, yield gap caused by water deficit; Yp, yield potential; Yw, water‐limited yields.

Scalar differences between the mean and median indicate high skewness and a deviation from normality. For the variable Ya, despite the proximity between the mean and median (3.00 and 3.06, respectively), the negative skewness coefficient (−1.30) and the K–S test confirm non‐normality. The variable Ygw demonstrates non‐normality, supported by statistical metrics such as high coefficients of variation, skewness, and kurtosis. These findings emphasize the importance of assessing data normality as a criterion for subsequent statistical and mathematical analyses (Soares et al., 2020).

### 3.2 Spatial patterns of soybean growing for Brazilian MRs

**FIGURE 2:**
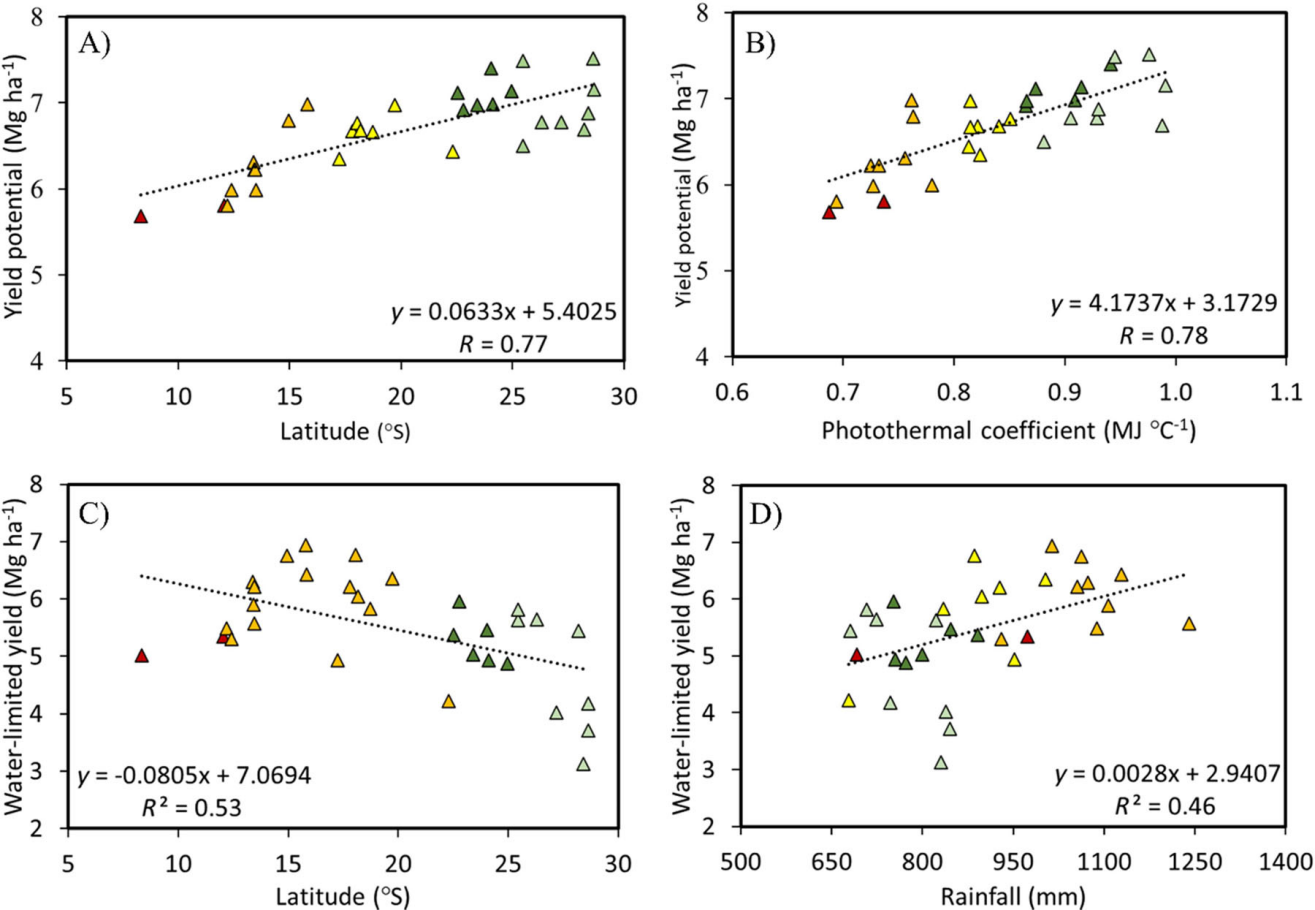
Relationship between (A) yield potential and latitude, (B) yield potential and photothermal coefficient, (C) water‐limited yield and latitude, and (D) water‐limited yield and rainfall for 32 locations in Brazil. Triangle colors indicate soybean macroregions: MR1 (light green), MR2 (dark green), MR3 (yellow), MR4 (orange), and MR5 (red).

Yp varied substantially among MRs. Southern macroregions (MR1 and MR2) recorded the highest Yp values, driven by favorable climate conditions and soil fertility, but also showed the largest Ygw, emphasizing the strong influence of rainfall variability and climatic events (Figure 2; Table 2). The Midwest (MR3) had the lowest Yp but exhibited relatively small water‐related Yg, reflecting more stable climatic conditions. Northern regions (MR4 and MR5) showed intermediate Yp values with moderate climatic constraints. These regional differences underscore the importance of tailoring management practices, including irrigation and sowing strategies, to local conditions.

**TABLE 2:** Nemenyi post hoc test results for non‐parametric Kruskal–Wallis *H* test.

Region	Yp	Yw	Ya	Ygw	Ygm
MR1	6.97^a^	4.70^c^	2.94^ab^	2.27^a^	1.75^b^
MR2	7.01^a^	4.98^bc^	3.07^a^	2.02^a^	1.91^b^
MR3	6.71^ab^	6.01^ab^	3.03^ab^	0.70^b^	2.98^a^
MR4	6.29^bc^	6.10^a^	3.07^a^	0.19^b^	3.03^a^
MR5	5.83^c^	5.16^abc^	2.60^ab^	0.67^b^	2.56^ab^

*Note*: Means followed by a common letter are not significantly different by the Nemenyi test at the 5% level of significance.

Abbreviations: MR1, macroregion 1; MR2, macroregion 2; MR3, macroregion 3; MR4, macroregion 4; MR5, macroregion 5; Ya, yield average; Ygm, yield gap caused by suboptimal crop management; Ygw, yield gap caused by water deficit; Yp, yield potential; Yw, water‐limited yields.

Yield potential was higher at higher latitudes (around 30° S), with the maximum observed in Cruz Alta/RS (7.5 Mg ha^−1^) at 28.7° S (Figure 2). Yp decreased progressively toward lower latitudes, with the minimum recorded in Baixa Grande do Ribeiro/PI (5.7 Mg ha^−1^) at 8.3° S. As shown in Figure 2A, for each degree increase in southern latitude, Yp increased by 0.063 Mg ha^−1^. The coefficient of determination (*R* ^2^) indicated a strong relationship between Yp and latitude (0.77), as well as with the photothermal coefficient (0.78).

The Yw ranged from 3.1 to 6.9 Mg ha^−1^ and decreased over the north‐south direction (Figure 2C), with the highest values in Campo Verde/MT (6.9 Mg ha^−1^), at 15.8° S, and the lowest in São Luiz Gonzaga/RS (3.1 Mg ha^−1^), at 28.4°S. In Figure 2D, it is observed that most places have rainfall above 800 mm; however, poor rainfall distribution penalizes yield in the south of the country. The variables latitude and rainfall showed a satisfactory R2 with Yw, being 0.53 and 0.46, respectively.

### 3.3 Yg: Water deficit and management

The differences between the MR's Yp, Yw, Ya, Ygw, and Ygm are presented in Table 2. The mean values were also tested with Kruskal–Wallis rank sum tests and Nemenyi tests. The Kruskal–Wallis test and Nemenyi post hoc test showed that there was an undeniable difference between regions for all variables observed. The Yp shows a significant increase from north to south in Brazil, with MR1 and MR2 showing the greatest potentials and showing no significant difference between them. For the Yw, all the MRs showed a significant difference according to the test, with the lowest Yw indicated for MR1 (4.70 Mg ha^−1^). The highest Yw were for MR3 and MR4, respectively (6.01 and 6.10 Mg ha^−1^) located in the Midwest region of Brazil.

The Ya for de MR's was separated into two groups by the Nemenyi post hoc test, with MR2 and MR4 showing higher yields (3.07 and 3.07 Mg ha^−1^), significantly differing from MR1, MR3, and MR5 (2.94, 3.07, and 2.60 Mg ha^−1^), with lower yields. As well as for Ya, Ygw separated MRs into two groups, however grouping MR1 and MR2 with the largest gaps (2.27 and 2.02 Mg ha^−1^) and MR3, MR4, and MR5 with the smallest gaps (0.70, 0.19, and 0.67 Mg ha^−1^). The MR3 and MR4 had the highest Ygm (2.98 and 3.03 Mg ha^−1^) with no significant difference between them. The smallest Ygm were observed for MR1 and MR2 (1.75 and 1.91 Mg ha^−1^), located further south. MR5 presented an intermediate Ygm (2.56 Mg ha^−1^), significantly different from all others.

The total Yg average observed in Brazil was 3.7 Mg ha^−1^, ranging from 2.7 Mg ha^−1^ in Gleba Celeste/MT, at the MR4, to 4.6 Mg ha^−1^ in São Luiz Gonzaga/RS, at MR1. As an example of the Yg variation between the locations, Campo Verde/MT (MR4) has the largest management gap with 3.8 Mg ha^−1^, and Cruz Alta/RS (MR1) has the smallest management gap with 0.8 Mg ha^−1^. The water gap was smaller in general, but the variation was considerably large showing values of 3.8 Mg ha^−1^ in Cruz Alta/RS, at the MR1, and values lower than 0.1 Mg ha^−1^ in four other locations at Mato Grosso state and in one location at Goias state, all at MR4.

**FIGURE 3:**
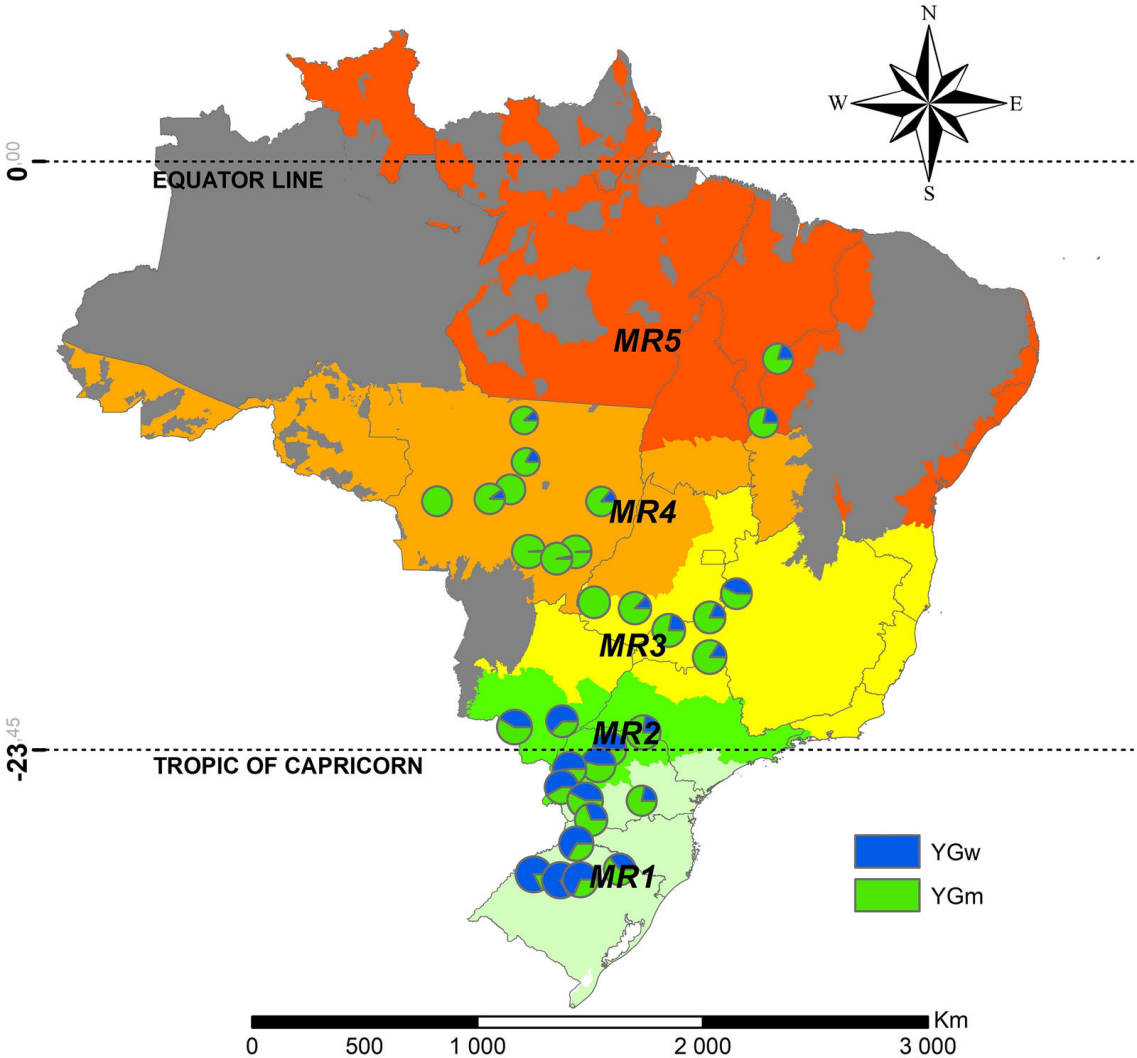
Yield gap represented by the size of the circles, separated into water‐limited (blue) and management‐related (green) yield gaps. Circle sizes are proportional to the relative contribution (%) of each component to the total yield gap for each location.

In Figure 3, the size of the circles represents the Yg, in which the largest gaps are in the South region with the largest circles on the map, and this is due to the existence of higher Yp (Tagliapietra et al., 2021). Also in the southern region, it was observed that the water deficit gap corresponds to >50% in most places. On the other hand, in the Midwest region of the country, >90% of the Yg corresponds to the management, due to the high amount and well‐distributed rainfall. There is a great difference is between the Ygw for MR1 and MR2, compared to the others, reaching >100%. Also Figure 3 demonstrates the high Yp values for these same MRs. The Ygw are 2.27 and 2.02 Mg ha^−1^, and this volume produced if irrigated is a significant addition for these regions.

### 3.4 Sowing date as a management strategy to yield increase

**FIGURE 4:**
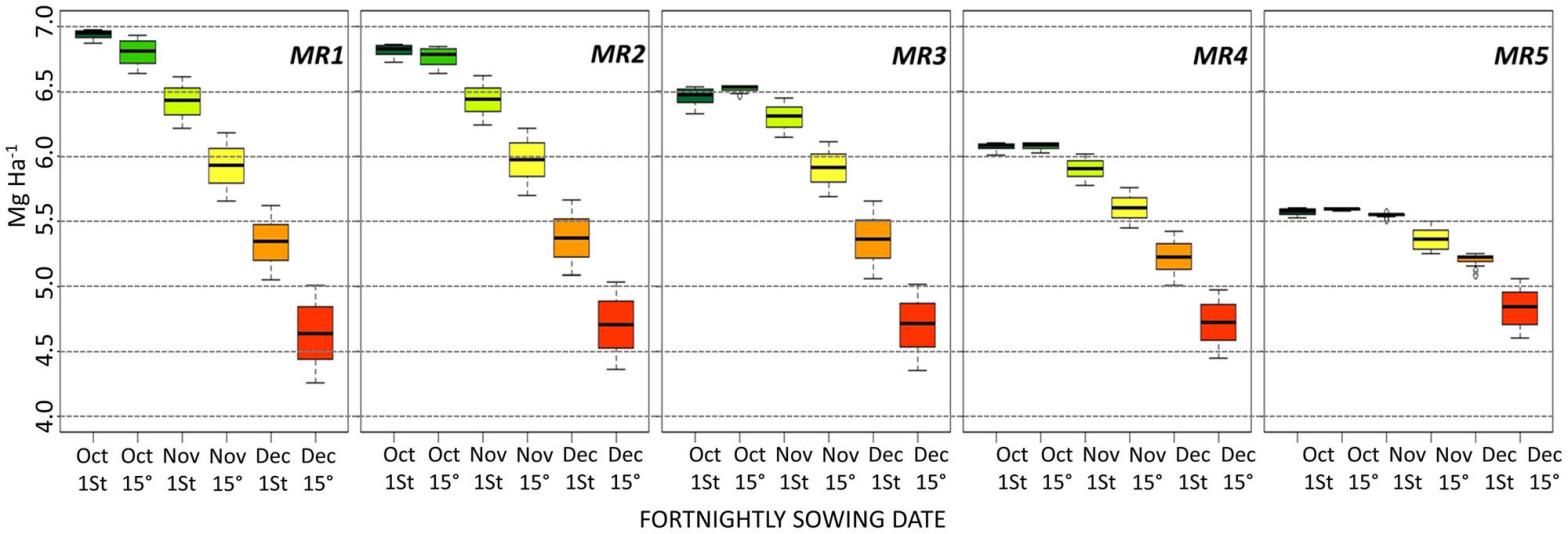
Yield potential (Mg ha^−1^) across sowing dates for each soybean macroregion in Brazil: Macroregion 1 (MR1) (A), Macroregion 2 (MR2) (B), Macroregion 3 (MR3) (C), Macroregion 4 (MR4) (D), and Macroregion 5 (MR5) (E). Boxplots represent simulated yield potential for fortnightly sowing dates from October 1 to December 15. Colors indicate yield magnitude, from higher yields (green) to lower yields (red). Yield values are expressed in megagrams per hectare (Mg ha^−1^).

Figure 4 corroborated the fact that MR1 and MR2 had high Yp. Furthermore, the boxplots demonstrated the yield reduction due to sowing delay for all five MRs. This yield reduction was more accentuated for MR1 and MR2. In MR1 (Figure 4A), the higher Yp reached 7.0 Mg ha^−1^ and began to decrease in October 30°, losing up to 42 kg ha^−1^ day^−1^. In MR2 (Figure 4B), the higher Yp reached 7.0 Mg ha^−1^ and started to decrease in second November, losing up to 42 kg ha^−1^ day^−1^. For MR3 (Figure 4C), Yp reached 6.7 Mg ha^−1^ and started decreasing in November 10°, losing up to 40 kg ha^−1^ day^−1^. In MR 4 (Figure 4D), the higher Yp reached 6.3 Mg ha^−1^ and began to decrease in November 11°, losing up to 33 kg ha^−1^ day^−1^. For MR5 (Figure 4E), Yp reached 5.8 Mg ha^−1^ and started to decrease in November 15°, losing up to 20 kg ha^−1^ day^−1^.

For MR3, MR4, and MR5, a downward Yp is observed in the best sowing window (6.71, 6.29, and 5.83 Mg ha^−1^), but even so, the same downward trend for the subsequent fortnights. For the MRs that are in regions with lower latitudes and warmer temperatures, there is a lower availability of solar radiation. Although the availability is lower, the variation is very small, implying smaller variations in Yp in sowing times.

### 3.5 Environmental and climatic factors that drives to yield variability

**FIGURE 5:**
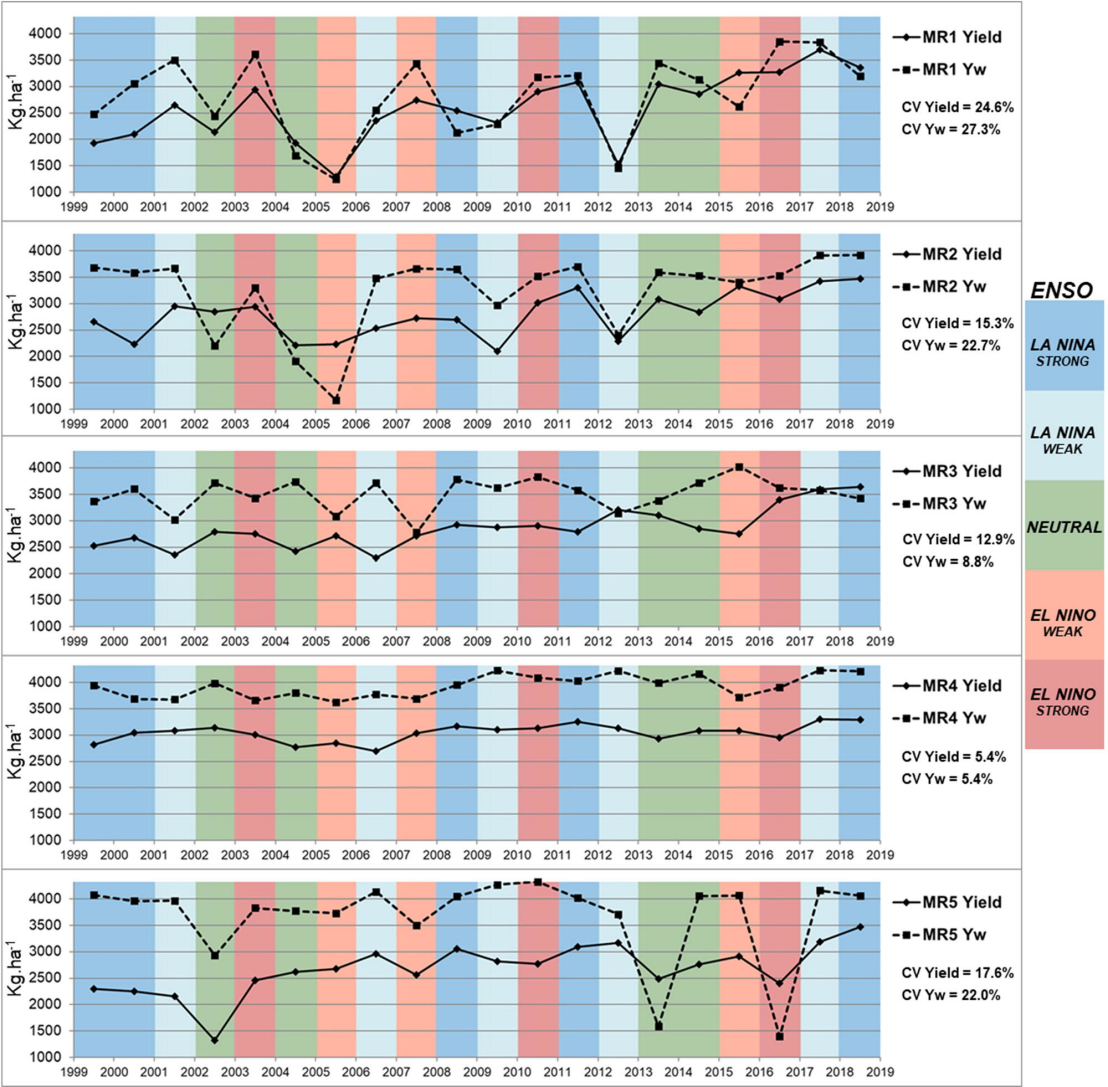
Twenty years of average yield and water‐limited yield potential across five macroregions of Brazil against annual registered El Niño‐Southern Oscillation (ENSO) climatic phenomenon.

Based on 20‐year data from 1999 to 2019 (Figure 5), soybean yields tend to decline during La Niña events in MR1 and MR2 in Brazil. This is due to extreme droughts associated with this phenomenon in southern South America (Berri et al., 2019; Nóia et al., 2020). In southern Brazil, it often leads to a decrease in rainfall and prolonged dry periods that significantly affect soybean crops. Even more, in MR1 and MR2, the main soybean fields are rainfed fields, where reduced precipitation significantly increases the risk of yield loss and even total crop loss, affecting overall soybean yields in these MRs.

The highest coefficients of variation (CV) observed for the 20‐year period for both real yield and Yw were for MR1 (24.6% and 27.3%). This indicates that the southernmost region is prone to very large interannual variations due to the unstable rainfall distribution. MR2 shows a similar pattern in terms of long‐term distribution but with relatively lower CVs for yield and Yw (15.3% and 22.7%).

Soybean productivity in Midwest Brazil, especially in MR3 and MR4, is not significantly affected by the ENSO phenomenon. For MR3, which is partially located in Midwest Brazil, the second lowest CVs for yield and Yw are observed (12.9% and 8.8%, respectively). MR4 has the lowest CVs (5.4% and 5.4%) for yield and Yw, respectively, and is located entirely in the Midwest region. For MR5, located in the extreme north of Brazil, the CVs for Ya and Yw are higher than for the Midwest MRs (17.6% and 22.0%).

## 4 DISCUSSION

### 4.1 Assessment Brazilian soybean yield potential and Yg

Brazil's vast territorial extent encompasses a wide range of environmental conditions and production systems. The combination of diverse climates, soil types, and management practices results in distinct yield profiles across the country. Accurate information on the spatial and temporal patterns of agricultural land use and yields across the Brazilian territory could improve the sustainability of Brazilian agriculture.

Such data can pinpoint regions where yield improvements can be achieved with minimal environmental impact (Marin et al., 2022). Using the standardized GYGA methodology (Van Ittersum et al., 2013), our analysis shows that the Yp of soybeans in Brazil exceeds that reported by Grassini et al. (2015) (4.4–7.1 Mg ha^−1^) and Rattalino Edreira et al. (2017) (5.4–6.1 Mg ha^−1^) in the United States. The Yw values (3.1–6.9 Mg ha^−1^) are also higher than those in Argentina (2.2–5.2 Mg ha^−1^) (Aramburu Merlos et al., 2015).

Yw variation among the different MRs producing soybeans is relatively low. This moderate variation in Yw (16.5%) among MRs likely leads to the high variation in Ygw (90.8%). This is since the model used to determine Ygw is extremely sensitive to variation in Yw, as it is a vector for variability in Ygw across soybean‐growing regions in Brazil. It is very important to note that Ygm is also quite high, although it is still classified as moderate (32.2%) according to Wilding and Drees classification.

A strong correlation was observed between Yp, latitude, and photothermal coefficient (*Q*), with Pearson coefficients of 0.77 and 0.78, respectively. This relationship is particularly pronounced in MR1, aligning with Zanon et al. (2016), who demonstrated that delayed sowing reduces Yp due to reduced solar radiation. Rainfall patterns are the primary drivers of Yw variability.

In the Midwest (MR4), the monsoon regime ensures rainfall coincides with critical growth stages (Grimm, 2003). Conversely, in southern Brazil, rainfall is distributed throughout the year but is less reliable during summer, increasing the risk of drought due to ENSO events (Arsego et al., 2018; Nóia et al., 2020). Ygw increases from north to south, reflecting more stable rainfall in tropical regions, a trend also observed globally (Saryoko et al., 2017). In contrast, Ygm decreases toward the south, where climatic factors dominate yield reductions, particularly in MR1 and MR2 (Battisti et al., 2018).

### 4.2 Water versus management in explaining soybean Yg

Yg are influenced by both climate risk and management practices. Even if a region has a high Yp due to environmental factors such as high availability of solar radiation, interannual heterogeneity in rainfall significantly increases the risk of production losses (Wang et al., 2021). This means that rainfed soybean production in locations where Yw and Ygw are high is unstable and may reach high levels in some years, while almost failing in others. In this case, an assessment of the Yg for different cropping areas is crucial to show the direction of public policies that stimulate and promote the practice of irrigation, especially in MRs with a higher risk of drought.

Improving soil management is another strategy to reduce drought impacts, particularly in southern Brazil. Practices that enhance soil water retention and root development can improve yield stability (Sentelhas et al., 2015). In MR3, MR4, and MR5, high Ygm values indicate significant opportunities to enhance agronomic practices. Improvements in soil fertility, cultivar selection, pest management, and other well‐established techniques could substantially increase yields. Ygm is the factor that can be improved most easily and quickly, as currently available management strategies can reduce this gap, affecting yield, profitability, and sustainability (Marin et al., 2022). From this perspective, it is extremely important to identify the Ygm at the spatial level for the different MRs as a decision‐making tool for the development of management strategies (Winck et al., 2023). The Ygm is equivalent to 67% of the Yg, which means that with the improvement of management practices, it is possible to increase the current yield by 82% and practically double Brazil's production by increasing it by 90 million tonnes of soybeans.

Soybean producers could improve the yield of their fields by adopting strategies to reduce Ygw, such as deepening the soil profile, which could increase Ya by 2.5 Mg ha^−1^ on average. When we consider the soil profile, we refer to practices to reduce soil compaction, soil acidity, and aluminum toxicity; control nematodes; and improve soil microbiota (Franchini et al., 2017). Other cropping practices are also important to close Yg, such as choosing the best genotype for the region (MG and growth habit), soil fertilization and seed inoculation with Bradyrhizobium (Barth et al., 2018), and crop protection to reduce pest and disease damage (Sentelhas et al., 2015).

Irrigation could be used during critical growing season phases and in years with short droughts. This strategy should be considered when Yp and the Yg due to water deficit are greater, as in the state of Rio Grande do Sul (MR1). Appropriate irrigation practices need to be developed to use available water resources more efficiently and achieve higher yields. High international soybean valuations and climatic variability in Brazil's main growing regions have increased demand for permits to establish new irrigated soybean fields, thereby increasing soybean yields in the tropics (Garcia y Garcia et al., 2010; Kuss et al., 2008). The Brazilian Water Agency reported that new permits were issued in 2014 to irrigate >28,000 ha of soybean acreage with central pivot systems.

A management method that can be adopted by farmers without causing an increase in costs is sowing within the Yp range, as previously described in Rio Grande do Sul (Tagliapietra et al., 2021; Zanon et al., 2016). The results show a decrease in Yp due to the delay in sowing date in all MRs of Brazil. In the south, Yp increases with early sowing due to higher solar radiation from autumn to summer, coinciding with the highest radiation rates in the reproductive phase. Controlling the sowing date could be a simple strategy to reduce Yg, but water deficit must be considered when sowing early, especially in seasons with high drought risk (Nóia et al., 2020).

Another sowing date consideration relates to the stability of modeled yield. For earlier sowing dates, the results for all MRs show much smaller and flatter fluctuations. This is a clear indication that in addition to a higher Yp for early sowing dates, stability is also much greater than for later sowing dates. This pattern was also observed by Nóia et al. (2020) for seasons with regular rainfall. For soybean in MR1 and MR2, which are more sensitive to periods of low rainfall early in the season, especially in La Nina years, one strategy could be to postpone sowing, as rainfall tends to increase from December. While this strategy means a reduction in Yp, it may protect some yield from the risk of drought, which could result in a total loss.

### 4.3 Interannual variability of soybean yields

Over the past two decades, soybean yields have shown considerable fluctuations, particularly in MR1 and, to a lesser extent, MR2. These variations closely mirror changes in Yw, which are largely driven by climatic events such as ENSO. During La Niña years, severe droughts lead to a marked reduction in Yw and, consequently, yields, a pattern also documented by Nóia et al. (2020). Historical analyses of the past 20 harvests in MR1 and MR2 further reveal that extreme droughts can also occur during El Niño Modoki events, as observed in 2005, one of the most severe drought years on record (Cavalcanti et al., 2015).

In the Midwest region of Brazil, which includes MR3 and MR4, soybean production remains stable despite ENSO occurrence. These MRs are the largest soybean‐producing areas in the country and benefit from favorable climatic conditions, such as well‐distributed rainfall and suitable temperatures (Battisti et al., 2018), allowing for consistent soybean productivity even during El Niño, La Ninã, or neutral events. Medium instability was indicated from MR5 for interannual soybean production. The results show a decrease in yield and Yw in El Niño years, indicating an inversion of the frequency of droughts in the southern regions (MR1 and MR2) (Grimm, 2003).

Different types of El Niño (canonical and Modoki) have distinct climate impacts, especially on rainfall patterns. The Modoki type, marked by central Pacific warming, has become more frequent and affects regions not typically influenced by canonical events, complicating agricultural planning. Distinguishing between ENSO types in real time remains challenging, highlighting the need for improved climate models (Marathe & Ashok, 2021). Even in the case of ENSO, there are multiple considerations regarding the occurrence of droughts, such as droughts in southern Brazil during La Niña years or in the north during El Niño years, as the phenomenon can vary not only in strength (strong vs. weak) but also in spatial configuration (Modoki vs. canonical). These variations directly affect the climatic conditions of each region (Cavalcanti et al., 2015; Yuan & Yamagata, 2015), indicating the need for more in‐depth studies across different soybean production zones in Brazil.

## 5 CONCLUSIONS

The highest Yp for soybean were observed in MR1 and MR2, followed by MR3, MR4, and MR5. Yw was greatest in the Brazilian Midwest and lowest in the southern regions. Each MR is characterized by distinct climatic and environmental conditions, leading to specific patterns of Yp, average yield, and Yw. These regional differences must be considered when designing strategies to close the exploitable Yg.

In MR1 and MR2, Yg are predominantly water‐limited due to frequent drought events. This underscores the need for targeted policies that support irrigation infrastructure and improve water use efficiency. Strategic investments in irrigation in these regions could substantially increase actual yields while reducing climate vulnerability. In MR3 and MR5, Yg are largely influenced by suboptimal management practices rather than water limitations. Therefore, initiatives that enhance technical capacity, expand extension services, and facilitate access to advanced technologies may be more effective in these areas. In MR4, where the water‐related Yg is minimal, yield improvements can be primarily achieved through better management, indicating a high return on investment in agronomic training and cultivar optimization.

Our findings also indicate that delayed sowing reduces Yp in all MRs, especially in the south, where the impact is stronger. This effect decreases over the season and across regions from MR1 to MR5 and is associated with greater yield heterogeneity. These results underline the importance of regionally adapted sowing calendars and the development of climate‐resilient cropping systems.

In addition, the interannual variability of yields is closely linked to ENSO. In MR1 and MR2, yield reductions occur more frequently in La Niña years, while in MR5, lower Yp and Yw occur in El Niño years. MR3 and MR4 show greater yield stability and are less sensitive to ENSO phases. These findings can guide the development of climate‐smart agricultural policies, including ENSO‐informed forecasting systems to optimize sowing dates and resource allocation at the MRal level.

Overall, this study provides a robust scientific foundation for differentiated public policies aimed at sustainable intensification of soybean production in Brazil. By tailoring investments in irrigation, management strategies, and climate risk mitigation to the specific constraints and opportunities of each MR, it is possible to enhance productivity while minimizing environmental impacts.

## AUTHOR CONTRIBUTIONS

**Mauricio Fornalski Soares**: Conceptualization; data curation; formal analysis; investigation; methodology; supervision; visualization; writing—original draft; writing—review and editing. **Gean Leonardo Richter**: Conceptualization; data curation; formal analysis; funding acquisition; investigation; methodology; project administration; resources; supervision; writing—original draft. **Nereu Augusto Streck**: Conceptualization; funding acquisition; investigation; project administration; supervision; validation; visualization; writing—review and editing. **Fabio Ricardo Marin**: Conceptualization; data curation; formal analysis; investigation; methodology; validation; writing—review and editing. **Evandro Henrique Figueiredo Moura da Silva**: Conceptualization; data curation; formal analysis; investigation; methodology; validation. **Eduardo Lago Taglieapietra**: Formal analysis; methodology; validation; visualization; writing—original draft; writing—review and editing. **José Eduardo Minussi Winck**: Conceptualization; investigation; validation; visualization; writing—original draft. **Michel Rocha da Silva**: Formal analysis; methodology; project administration; visualization; writing—original draft. **Felipe Schmidt Dalla Porta**: Supervision; visualization; writing—review and editing. **Júlia Farias**: Data curation; validation; visualization; writing—original draft; writing—review and editing. **Alencar Junior Zanon**: Conceptualization; data curation; funding acquisition; project administration; resources; supervision; visualization.

## CONFLICT OF INTEREST STATEMENT

The authors declare no conflicts of interest.

## Supporting information



Supplemental Material
