# Supplementary material for: Assessing environmental and management factors that drive soybean yield gaps in Brazil
Source: J Environ Qual. 2025 Sep 9;54(6):1383–96. doi: 10.1002/jeq2.70076 (PMC12593302; doi:10.1002/jeq2.70076)
Supplement: Supplementary file 1 — Supplemental Material [file JEQ2-54-1383-s001.docx]

Supplemental Material

The supplementary material includes the identifications of the weather stations used to run the DSSAT-CROPGRO model. The input data listed are: Sowing date, relative maturity group (MG), plant population, soil types and predominance in percentage.

| OBJECTID | Name | IC | City | State | Sowing date | MG |
| --- | --- | --- | --- | --- | --- | --- |
| 1 | yH_32_soy | NADA | Baixa Grande do Ribeiro | PI | 15-october | 9.0 |
| 2 | yH_29_soy | YH29 | Brasilandia do Sul | PR | 05-october | 6.5 |
| 3 | yH_15_soy | CA01 | Campo Mourão | PR | 05-october | 6.5 |
| 4 | yH_06_soy | CA02 | Campo Verde | MT | 15-october | 8.0 |
| 5 | yH_04_soy | CA03 | Canarana | MT | 05-november | 8.0 |
| 6 | yH_09_soy | CA04 | Capinópolis | MG | 15-october | 7.0 |
| 7 | yH_10_soy | CA05 | Catalão | GO | 15-october | 8.0 |
| 8 | yH_19_soy | CR06 | Cruz Alta | RS | 10-october | 6.0 |
| 9 | yH_02_soy | GL07 | Gleba Celeste | MT | 15-october | 8.0 |
| 10 | yH_21_soy | IB08 | Ibirubá | RS | 15-october | 5.5 |
| 11 | yH_17_soy | IR09 | Iraí | RS | 10-october | 6.0 |
| 12 | yH_16_soy | IR10 | Irati | PR | 05-november | 6.0 |
| 13 | yH_13_soy | IV11 | Ivinhema | MS | 15-october | 6.5 |
| 14 | yH_20_soy | LA12 | Lagoa Vermelha | RS | 01-november | 5.5 |
| 15 | yH_31_soy | YH31 | Lucas do Rio Verde | MT | 15-october | 8.0 |
| 16 | yH_14_soy | MA13 | Maringá | PR | 05-october | 6.5 |
| 17 | yH_23_soy | YH23 | Mineiros | GO | 15-october | 7.0 |
| 18 | yH_08_soy | PA14 | Paracatu | MG | 15-october | 7.0 |
| 19 | yH_24_soy | YH24 | Pato Branco | PR | 05-november | 6.0 |
| 20 | yH_12_soy | PO15 | Ponta Porã | MS | 15-october | 6.5 |
| 21 | yH_05_soy | PO16 | Poxoréo | MT | 15-october | 8.0 |
| 22 | yH_26_soy | YH26 | Primavera do Leste | MT | 15-october | 8.0 |
| 23 | yH_28_soy | YH28 | Quedas do Iguaçu | PR | 05-october | 6.5 |
| 24 | yH_07_soy | RI17 | Rio Verde | GO | 15-october | 7.0 |
| 25 | yH_22_soy | YH22 | Santa Carmem | MT | 15-october | 8.0 |
| 26 | yH_03_soy | SA18 | São José do Rio Claro | MT | 05-october | 8.0 |
| 27 | yH_18_soy | SA19 | São Luiz Gonzaga | RS | 10-october | 6.0 |
| 28 | yH_27_soy | YH27 | São Pedro do Iguaçu | PR | 05-october | 6.5 |
| 29 | yH_30_soy | YH30 | Sapezal | MT | 15-october | 8.0 |
| 30 | yH_01_soy | TA20 | Taguatinga | TO | 03-november | 8.0 |
| 31 | yH_25_soy | YH25 | Tarumã | SP | 05-october | 6.5 |
| 32 | yH_11_soy | UB21 | Uberaba | MG | 15-october | 7.0 |

| Plant population | Soil - SiBCS* | Soil taxonomy | %Soil |
| --- | --- | --- | --- |
| 300000 | Latossolo amarelo distrófico | Oxisols | 62.3% |
| 275000 | Argissolo acinzentado distrofico | Ultisols | 39.9% |
| 275000 | Nitossolo | Alfisols | 21.8% |
| 300000 | Latossolo vermelho distrófico | Oxisols | 19.4% |
| 250000 | Latossolo vermelho-amarelo distrófico | Oxisols | 44.0% |
| 280000 | Latossolo vermelho distrófico | Oxisols | 40.6% |
| 280000 | Latossolo vermelho distrófico | Oxisols | 47.6% |
| 300000 | Latossolo vermelho alico | Oxisols | 23.9% |
| 300000 | Latossolo vermelho-amarelo distrófico | Oxisols | 95.5% |
| 250000 | Latossolo vermelho alico | Oxisols | 69.3% |
| 270000 | Latossolo vermelho alico | Oxisols | 55.2% |
| 250000 | Latossolo vermelho distrófico | Oxisols | 15.4% |
| 300000 | Latossolo vermelho distrófico | Oxisols | 47.3% |
| 250000 | Latossolo amarelo distrófico | Oxisols | 17.0% |
| 300000 | Latossolo vermelho-amarelo distrófico | Oxisols | 85.7% |
| 300000 | Argissolo acinzentado distrofico | Ultisols | 28.9% |
| 300000 | Latossolo vermelho distrófico | Oxisols | 30.4% |
| 280000 | Latossolo vermelho-amarelo distrófico | Oxisols | 29.7% |
| 250000 | Latossolo amarelo distrófico | Oxisols | 11.8% |
| 325000 | Latossolo vermelho alico | Oxisols | 73.1% |
| 300000 | Latossolo vermelho distrófico | Oxisols | 23.3% |
| 300000 | Latossolo vermelho distrófico | Oxisols | 22.9% |
| 275000 | Nitossolo | Alfisols | 22.1% |
| 300000 | Latossolo vermelho alico | Oxisols | 31.1% |
| 300000 | Latossolo vermelho-amarelo distrófico | Oxisols | 98.3% |
| 300000 | Latossolo vermelho-amarelo distrófico | Oxisols | 72.9% |
| 300000 | Latossolo vermelho alico | Oxisols | 63.3% |
| 275000 | Nitossolo | Alfisols | 26.5% |
| 300000 | Latossolo vermelho-amarelo distrófico | Oxisols | 48.6% |
| 230000 | Latossolo amarelo distrófico | Oxisols | 33.5% |
| 300000 | Argissolo vermelho-amarelo eutrofico | Ultisols | 48.7% |
| 280000 | Latossolo vermelho alico | Oxisols | 29.5% |
|  |  |  |  |
| MG - Maturity group | |  |  |
| **SiBCS - Brazilian soil classification system* | |  |  |
